# Use of Multivariate Adaptive Regression Splines (MARS) and Classification and Regression Tree (CART) Data Mining Algorithms to Predict Live Body Weight of Tswana Sheep

**DOI:** 10.3390/biology14111516

**Published:** 2025-10-30

**Authors:** Monosi Andries Bolowe, Lubabalo Bila, Ketshephaone Thutwa, Patrick Monametsi Kgwatalala

**Affiliations:** 1 Department of Animal Sciences, Botswana University of Agriculture and Natural Resources, Private Bag, Gaborone 0027, Botswana; kthutwa@buan.ac.bw (K.T.); pkgwatal@buan.ac.bw (P.M.K.); 2 Department of Animal Production, Potchefstroom College of Agriculture, Potchefstroom 2520, South Africa; bilalubabalo94@gmail.com

**Keywords:** body weight, correlation, cross-validation, goodness of fit

## Abstract

This study was conducted to (i) determine the association between live body weight (BW) and biometric traits, (ii) examine the effect of biometric traits on BW of Tswana sheep using MARS and CART data mining algorithms, (iii) compare the performance of the algorithms and, finally, select the best algorithm for predicting BW in Tswana sheep. BW and sixteen biometric traits were measured from 392 Tswana sheep (males = 85 and females = 307) aged three to four years. Pearson’s correlation coefficients were used to establish the relationship between BW and biometric traits. The goodness of fit criteria were computed to assess the predictive performance of the data mining algorithms and select the best-fit model for predicting BW. The results showed that BW had a positive and significant correlation with heart girth (HG) (r = 0.99); thus, HG was used as a sole predictor of BW. The goodness of fit results indicated that MARS has a higher predictive performance than the CART algorithm, suggesting that the MARS algorithm can be used to predict BW Tswana sheep. These findings are an important statistical tool for the selection and concurrent improvement of useful biometric traits in genetic improvement programs to improve BW in Tswana sheep.

## Simple Summary

Indigenous Tswana sheep play an important role in household food security and socio-cultural obligations for resource-poor farmers. For these farmers, weighing scales are not readily accessible; hence, sales of sheep are mostly dependent on physical appearance. Therefore, as was the purpose for this study, determining the relation between body weight (BW) and other linear body measurements and using multivariate adaptive regression splines (MARS) and classification and regression tree (CART) data mining algorithms to predict BW in Tswana sheep is key for resource poor farmers, particularly in places where there is a lack of weighing scales. Heart girth (HG) showed strong and significant correlations with BW. However, the correlation does not show the influence of HG on BW; hence, MARS and CART were used to determine the effect of HG on the BW of Tswana sheep. The MARS algorithm was the easier and more precise algorithm to use in predicting BW in Tswana sheep than the CART model. The high correlation of heart girth and body weight could also be used as an indirect selection criterion, as selecting for sheep with larger heart girth would result in a concurrent improvement in body weight in Tswana sheep, leading to increased meat production.

## 1. Introduction

The Tswana sheep is a sheep breed native to Botswana and is characterized by a short fat tail, a medium-sized body, and high adaptability to local endemic conditions [1]. Tswana sheep are primarily kept for mutton production and can be sold to generate household income; they are a significant contributor to the socio-economic and cultural livelihoods of people in the rural populace [1]. In the traditional setup, where finding weighing scales is challenging [2], the sale and purchase of animals typically rely on their physical appearance rather than their actual body weight (BW). As a result, farmers do not receive the actual price for their animals’ worth [3]. This is despite BW being regarded as the base of selling. Several scholars across various parts of the world argue that BW is a particularly important trait of economic importance in livestock farming because sales are directly dependent on the animal’s BW [4,5,6]. Again, the live BW of animals in the flock is particularly important in terms of determining breeding strategies and herd management [7]. For instance, knowing live BW is key in calculating the optimum feed amount per sheep, determining market price more reliably, and determining drug dosage [8,9]. Therefore, predicting live BW in farm animals is a perfect alternative and solution in instances where weighing scales are not readily available [10]. Furthermore, according to [11], animal breeders have now shifted their research attention to enlightening the association between live BW and other linear morphometric traits to predict live BW [2] and, consequently, improving meat production [11]. This is because linear body measurements have a direct correlation with live BW, a phenomenon that has been used to predict live BW from linear body measurements using simple and multiple regression techniques [3,12].

However, the prediction of live BW using regression techniques is affected by multicollinearity problems that arise when there is a high correlation among body measurements [13,14]. Faraz et al. reported that the establishment of multivariate adaptive regression splines (MARS) and classification and regression tree (CART) data mining algorithms overcomes the multicollinearity problems in predicting live BW [11]. Fatih et al. defined the MARS data mining algorithm as a non-parametric regression technique that does not require any speculation about the distribution and correlation of variables entered in the predictive model to be built into statistical evaluation [2]. MARS has been used in predicting the body weight of different livestock species, including camels [2], cattle [5], goats [6,14], and sheep [15,16]. CART, on the other hand, is a recursive partitioning method that can predict both the categorical dependent variable (classification) and continuous dependent variable (regression) by building trees [17]. CART is regarded as a great geometric data mining algorithm that assesses the most imperative parameters in a specific data set and aids in the design of a specific model [2]. CART has been used in estimating body weight of different domesticated animals, including sheep [7,11,17], indigenous South African goats [9], and South African Sussex cattle [5].

The importance of developing live BW prediction tools for livestock farmers, particularly in the rural populace where weighing scales are unavailable, cannot be overemphasized. However, to the best of our knowledge, there is no well-known literature on the prediction of live BW from the linear body measurements of indigenous Tswana sheep using MARS and CART data mining algorithms. Hence, the objectives of this study were to (1) determine the association between live BW and linear body measurements of indigenous Tswana sheep using correlation analysis, (2) to determine the effect of linear body measurements on the live BW of indigenous Tswana sheep using MARS and CART data mining algorithms, and (3) select the best predictive algorithm. This study will be helpful to native Tswana sheep farmers in the selection and concurrent improvement of useful linear body measurements in genetic improvement programs to improve BW.

## 2. Materials and Methods

### 2.1. Study Site

**Figure 1:**
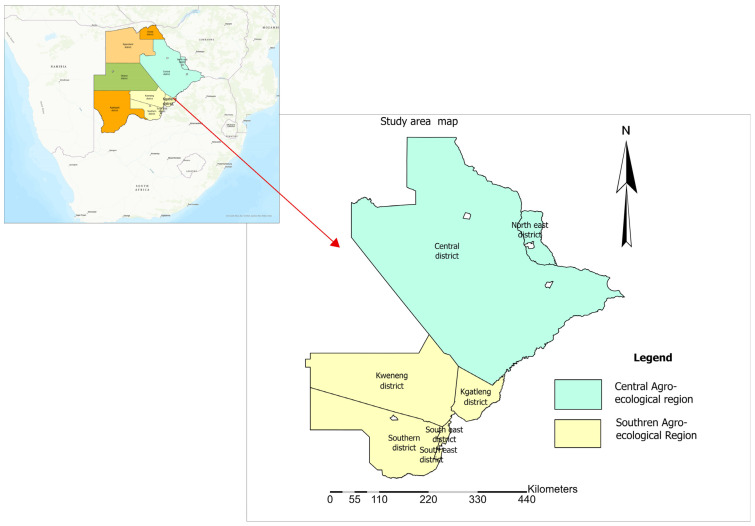
Map showing the geographical location of the study areas.

The study was conducted in the Southern and Central agroecological regions of Botswana (Figure 1), the only two regions with indigenous Tswana sheep populations. The Southern region, made up of Kweneng, Kgatleng, Southern, and the South-East districts, stands at 3002 ft above sea level with coordinates 22°00′ S and 26°00′ E. The region covers a total area of 74,100 km^2^ with an annual precipitation of around 550 mm, which also occurs during the summer seasons between November and May. The Southern region is classified as a hardveld savanna, characterized by tall grasses, bushes, and trees. The Central region, made up of the Central and North-East districts, also stands at 3002 ft above sea level, with coordinates 22°00′ S and 26°00′ E, and covers a total area of 147,730 km^2^. Annual precipitation is around 650 mm and is experienced mostly during the summer season from November to May. This region’s geographical landscape is dominated by mountainous, sandy, and savanna woodland, particularly *colophospermum mopane* trees.

### 2.2. Source of Animal and Management

Sampled indigenous Tswana sheep in the hands of farmers in the study regions were used as a source of animals. The animals used for this study were kept under the traditional grazing management system, where animals are allowed to freely graze in communal areas during the day and afternoon. Animals are normally watered once to twice a day, and the animals receive herd health management practices, which is typical of the region.

### 2.3. Sampling Methods

The country was demarcated into four agroecological regions: the Central (made up of Central and North-East districts), Southern (constituted by Kweneng, Southern, South-East, and Kgatleng districts), Ngamiland (made up of Chobe and North-West districts), and Ghanzi (made up of Ghanzi and Kgalagadi districts) regions. However, during data collection, it was realised that Tswana sheep are only found in two agroecological regions, the Southern and Central agroecological regions. These were thus the study areas. Firstly, in each region, discussions were held with regional agricultural officers in the Department of Veterinary Services to establish the distribution of indigenous Tswana sheep in the regions. Then, a multi-stage purposive sampling technique was employed first to select ten geographically distant and ecologically isolated villages with traditionally recognized indigenous Tswana sheep populations. A systematic random sampling technique was then used to select 5 households. From each household, in the Southern agroecology, a purposive random sampling technique was employed to select only 5 unrelated Tswana sheep, whereas, in the Central agroecology, on average, 3 unrelated Tswana sheep were selected for qualitative and quantitative trait recording, depending on flock size, to ensure the phenotypic distinctiveness of the sheep sampled.

In the sampled localities, statistics from local veterinary agents showed that the population of Tswana sheep adds up to 19,578, using the following formula:

$$n=\frac{N}{1+N{\left(e\right)}^{2}}$$

where

n = the required sample size,
N = the population size,
e = the acceptable error of estimation (0.05).

The sample size used was calculated as follows:

$$n=\frac{19,578}{1+19,578{\left(0.05\right)}^{2}}=392$$

The sample size in this study (392) was also guided by the literature papers in this subject. For instance, a study by [5] on the use of data mining algorithms to predict BW of South African Sussex cattle at weaning and [6] on the use of data mining algorithms to predict BW of Dorper sheep used a sample size of 101 and 242, respectively. These sample sizes were far below the sample size used in this study.

### 2.4. Data Collection Procedures

**Figure 2:**
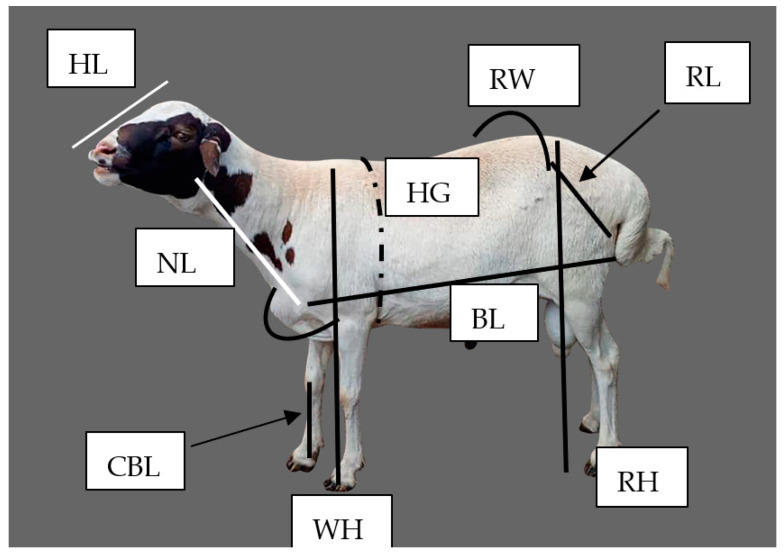
Schematics of morphometric traits in sheep. HG = heart girth, BL = body length; SW = shoulder width; NL = neck length; WH = wither height; RH = rump height; HW = head width; HL = head length; EW = ear width; EL = ear length; CBC = cannon bone circumference; CBL = cannon bone length; RL = rump length; RW = rump width; TL = tail length; TC = tail circumference; SC = scrotal circumference.

The body weight and linear body measurements of each animal sampled were recorded from 392 Tswana sheep (48 and 37 males in the Southern and Central regions, respectively, and 173 and 134 females in the Southern and Central regions, respectively). Pregnant ewes were excluded from the study to avoid the influence of pregnancy on biometric measurements. Measurements were made following the breed morphological features descriptor guidance list of FAO (2012). Quantitative traits (heart girth (HG), body length (BL), wither height (WH), rump width (RW), ear length (EL), tail length (TL), tail circumference (TC), head length (HL), head width (HW), shoulder width (SW), cannon bone length (CBL), cannon bone circumference (CBC), neck length (NL), rump length (RL), rump height (RH), and scrotal circumference (SC) (in males)) were measured using a flexible tailor’s measuring tape, with records taken to the nearest centimetres (cm). Body weight was measured using a suspended spring balance with a 110 kg capacity. All measurements were taken early in the morning before animals were released for grazing and drinking to avoid the effect of feeding and watering impacting the animals’ body weight. Schematics of the quantitative traits measured are shown in Figure 2.

The animals were restrained in an upright, unforced plane position during data collection. All measurements were taken by the same personnel during the study period for consistency. Pregnant ewes were excluded from sampling because pregnancy influences body measurements. Each animal sampled was identified by its sex and sampling site (agroecological region). Sex was characterized as male and female.

### 2.5. Statistical Analysis

#### 2.5.1. Descriptive Statistical Analysis

The Generalized Linear Model procedures (Proc glm) of the Statistical Analysis System (SAS release 9.1, 2003) were used to analyze the quantitative data to examine the influence of sex on body weight and linear body measurements. The model used for the least square mean analysis of BW and other linear body measurements in ewes, castrates, and rams, except scrotal circumference, was as follows:

Yij = µ + Si + eij

where

Yij = Body weight or linear body measurement,
µ = overall mean,
Si = the fixed effect of the ith sex (i = male, female),
eij = random residual error.

Pearson’s correlation coefficients for each trait under study were estimated between live body weight and other linear body measurements (LBMs) for each sex using the correlation procedure (PROC CORR) of SAS release 9.1 2003 to establish the direction and strength of the relationships between live body weight (response variable) and other LBMs (explanatory variables). R-studio using the EhoGof package (version 0.1.1) was used to run the data mining algorithms. Decision tree algorithms were used to design the model to estimate BW from the linear body measurements of indigenous Tswana sheep according to [13]. A ten-fold cross-validation resampling method was employed for the CART data mining algorithm as recommended in [18]. Furthermore, the goodness of fit test criteria were used to assess the predictive performance of MARS and CART algorithms and, consequently, select the best data mining algorithm for estimating BW from linear body measurements in indigenous Tswana sheep.

#### 2.5.2. Multivariate Adaptive Regression Spline (MARS) Algorithm

MARS is a non-parametric regression method developed by the author of [19] for handling pattern recognition problems in regression and classification for non-linear data. In the current study, the MARS algorithm was conducted as explained in [20,21], and its prediction equation can be written as follows:

$$f\left(x\right)=\beta_{0}+\sum_{m=1}^{m}\beta_{m}\lambda_{m}\left(x\right)$$

where *f(x)* is the expected response, β_0_ and β_m_ are parameters calculated to give the best data fit (intercept), and m is the number of basic functions (BFs) in the model. In the MARS model, the basis function was made up of a single univariable spline function or a combination of more than one spline function for diverse predictor inputs. The MARS data mining algorithm, BF, λ_m_(x), can thus be defined as follows:

$$f\left(x\right)=\beta_{0}+\Sigma_{m=1}\beta_{m}\Pi_{k=1}h_{m}\left(x_{v\left(k,m\right)}\right)$$

where *f(x)* denotes the estimated value of the dependent variable, β_0_ and β_m_ are the intercept, and $h_{m}\left(x_{v\left(k,m\right)}\right)$ is the basis function, whereas v(k,m) is the index of the predictor for the mth component of the kth product, and K is the parameter regulating the order of interaction. After building the most suitable MARS model, basic functions that had a low contribution to the model fitting performance were removed in the pruning process based on the generalized cross-validation error (GCV) [20,21].

$$GCv\left(\lambda \right)=\frac{\sum_{1=1}^{n}{\left(y_{i}-y_{jp}\right)}^{2}}{{\left(1-\frac{m\left(\lambda \right)}{n}\right)}^{2}}$$

where n is the number of training cases, y_i_ is the observed response variable, y_ip_ is the estimated value of the response variable, and M(λ) is a penalty function for the complexity of the model with λ terms.

#### 2.5.3. Classification and Regression Tree (CART) Algorithm

CART is a repetitive algorithm tree that is built up by dividing a node into pairs of child nodes repetitively, beginning with the root node that contains the whole sample under study according to [22] suggestions. CART is a type of modelling where the dependent variable is binary, that is, it can either be true or false; a one or a two; a yes or a no. It is usually one of the three. CART encompasses a decision and a classification tree in that a decision tree makes a statement and ascertains if it is true or not. If the decision tree classifies things into categories, it is called a classification tree, and when the decision tree predicts numeric values, it is then referred to as a regression tree. Therefore, the CART data mining algorithm encompasses both classification and prediction analysis.

#### 2.5.4. Goodness of Fit Test

The goodness of fit test criteria were used to calculate and select the best model between the simple linear regression model, MARS, and CART according to [18]. The following goodness of fit test criteria were computed for training and testing the datasets:

Pearson’s correlation coefficient (r)

$$r=\frac{\mathrm{cov}\left(y_{i},y_{ip}\right)}{s_{yi}s_{yip}}$$

Relative root-mean-square error (RRMSE)

$$RRMSE=\sqrt{\frac{\frac{1}{n}\sum_{i=1}^{n}\;\left(yi^{-y}\right)\times 2}{\bar{y}}}$$

Mean error (ME)

$$ME=\frac{1}{n}\sum_{i=1}^{n}\left(y_{i}-y_{ip}\right)$$

Coefficient of determination (Rsq)

$$Rsq=1-\frac{\sum_{i=1}^{n}{\left(y_{i}-{\hat{y}}_{i}\right)}^{2}}{\sum_{i=1}^{n}{\left(y_{i}-\bar{y}\right)}^{2}}$$

Adjusted coefficient of determination (ARseq)

$$ARseq=1-\frac{\frac{1}{n-k-1}\sum_{i=1}^{n}{\left(y_{i}-{\hat{y}}_{i}\right)}^{2}}{\frac{1}{n-1}\sum_{i=1}^{n}{\left(y_{i}-\bar{y}\right)}^{2}}$$

Performance index (PI)

$$PI=\frac{rRMSE}{1+r}$$

Root-mean-square error (RMSE)

$$RMSE=\sqrt{\frac{1}{n}\sum_{1=1}^{n}{\left(y_{i}-\hat{y}i\right)}^{2}}$$

Standard deviation ratio

$$SDR=\sqrt{\frac{\frac{1}{n-1}\sum_{i=1}^{n}{\left(\varepsilon_{i}-\bar{\varepsilon }\right)}^{2}}{\frac{1}{n-1}\Sigma_{1=1}^{n}{\left(y_{i}-\bar{y}\right)}^{2}}}$$

Mean absolute percentage error (MAPE)

$$MAPE=\frac{1}{n}\sum_{i=1}^{n}\left|\frac{y_{i}-{\hat{y}}_{i}}{y_{i}}\right|\times 100$$

Mean absolute deviation (MAD):

$$MAD=\frac{1}{n}\sum_{i=1}^{n}\left|\frac{y_{i}-{\hat{y}}_{i}}{y_{i}}\right|$$

Akaike information criteria (AIC)

$$AIC=N\;Ln\left(\frac{SSE}{N}\right)+2p$$

Relative approximation error (RAE)

$$RAE=\sqrt{\frac{\sum_{i=1}^{n}{\left(y_{i}-{\hat{y}}_{i}\right)}^{2}}{\sum_{i=1}^{n}y\frac{2}{i}}}$$

Coefficient of variation (CV)

$$CV=\frac{SD}{Mean}\times 100=\frac{\sqrt{\frac{1}{n-1}\sum_{i=1}^{n}{\left(\varepsilon_{i}-\bar{\varepsilon }\right)}^{2}}}{\bar{y}}\times 100$$

## 3. Results

### 3.1. Descriptive Statistics

The overall descriptive (mean, standard deviation, minimum, and maximum) of BW and linear body measurements for Tswana sheep are presented in Table 1.

The descriptive statistics (mean ± SE) of BW and linear body measurements for Tswana sheep as affected by sex are presented in Table 2. Males had significantly (*p* < 0.05) higher values in BW and most linear body measurements than females. Males and females did not significantly (*p* > 0.05) differ in BL, NL, EW, EL, and CBL. There were no significant differences (*p* > 0.05) in BW and most morphometric traits between males and castrates except TL and TC.

### 3.2. Pearson’s Correlation Coefficients Between BW and Linear Body Measurements

Pearson’s correlation coefficients between live BW and morphometric traits for indigenous Tswana rams and ewes are presented in Table 3. In rams, live BW had strong positive and significant (*p* < 0.05) correlations with HG (0.99), BL (0.83), SW (0.72), WH (0.80), and RH (0.85) but had moderate positive and significant correlations with HL (0.49), CBL (0.46), NL (0.53), RL (0.66), and RW (0.52). Live BW also had low, weak, and insignificant (*p* > 0.05) correlations with HW (0.38), EW (0.12), EL (0.095), CBC (0.009), TL (.21), and TC (0.20). In ewes, live BW was strongly, positively, and significantly (*p* < 0.05) correlated with HG (0.99), BL (0.79), WH (0.80), RH (0.77), and NL (0.72), but had moderate positive and significant correlations with SW (0.66), NL (0.58), RL (0.57), and RW (0.61). Ewes also showed low, weak, and insignificant correlations between BW and HW (0.23), HL (0.38), EW (0.091), EL (0.19), CBC (0.10), CBL (0,38), TL (0.18), and TC (0.11). With regards to castrates, live BW had strong, positive, and significant (*p* < 0.05) correlations with HG (0.99) and BL (0.77) but had moderate correlations with RH (0.41), TC (0.44), CBC (0.41), CBL (0.56), RL (0.57), and RW (0.63) (Table 4). Castrates, however, showed weak correlations between BW and NL (0.12), HW (0.01), HL (0.20), EW (0.35), EL (0.25), WH (0.32), and TL (0.20).

### 3.3. MARS Model Outcome

The results of the MARS model are presented in Table 5. The final BW has been explained with two basic functions in the MARS prediction model. Two basic functions (BFs) from the MARS model, all with one single order term variable, were discovered and had an intercept coefficient of 46.32. On the first BF, HG had a cut-point of 84 cm for a negative coefficient of −1.11, whereas in the other BF, HG had a positive coefficient of 1.81. Briefly, this indicates that MARS described the influence of linear body measurements with both negative and positive coefficients on BW. The influence of the BW of Tswana sheep was in the positive direction when HG > 84 cm, with a coefficient of 1.80, and the influence was in the negative direction when HG < 84 cm, with a coefficient value of −1.11.

The MARS model above appears to be a simple model with only one predictor. The question that arises, therefore, is whether it is necessary to use complex modelling algorithms with one predictor. A stepwise regression was employed to ascertain if a simple linear regression would not be sufficient in predicting BW compared to machine learning algorithms. The results, as per the goodness of fit (Table 6), indicate that not only does MARS have a better predictive performance than simple linear regression, but it also gives a cut-off point at which HG begins to influence BW in Tswana sheep, something which linear regression is unable to depict.

### 3.4. CART Model

The findings of the CART data mining algorithms based on the cross-validation technique are presented in Table 7. The calculated algorithms produced a tree structure with six terminal nodes with the smallest relative error (the cross-validation error of 0.04) and mean of the error (0.05), indicating that cross-validation and the coefficient of determination were close to each other.

**Figure 3:**
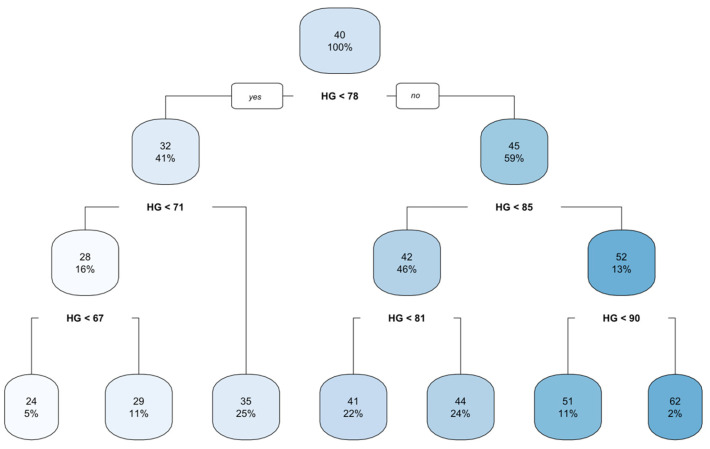
A regression tree diagram constructed using the CART algorithm. HG = heart girth; yes is always on the left; no is always on the right.

The regression tree created using the CART decision tree algorithm for predicting BW from linear body measurements in mature indigenous Tswana sheep is shown in Figure 3. The tree produced six terminal nodes. HG was found to be the significant sole independent variable for predicting BW in Tswana sheep. According to the top root node, the overall live BW of all Tswana sheep under study was 40 kg. From the base node, using HG and 78 cm, the analysis split Tswana sheep in the current study into smaller subgroups. In the first subgroup, when HG < 78 cm, the average BW determined was 32 kg in 41% of the flock; if HG > 78 cm, the average BW was 45 kg as determined in 59% of the flock. Using HG and 71 cm, the average BW was 28 kg, accounting for 16% of the flock; however, when HG > 71 cm, the average BW of Tswana sheep was determined to be 35 kg in 25% of the flock. When HG < 68 cm, the average BW determined was 24 kg in 5% of the flock, whereas when HG > 68 cm, the average BW was 29 kg as determined in 11% of the flock. When HG < 81 cm, the average BW was 41 kg in 22% of the flock, whereas when HG > 81 cm, the average BW determined was 44 kg in 24% of the flock. When the HG was less than 90 cm, the mean BW of Tswana sheep was 51 kg as determined in 11% of the flock, while the average BW was 62 kg in only 2% of the flock when the HG was greater than 90 cm.

### 3.5. Comparison of MARS and CART Data Mining Algorithms

The goodness of fit criteria were employed to assess and compare the predictive performance and consequential selection of the best algorithm (between MARS and CART) for estimating BW in Tswana sheep. The results are summarized in Table 8. MARS data mining algorithm revealed smaller RMSE, RRMSE, SDR, CV, RAE, MAPE, MAD, IP, AIC, and greater r, RSq, and ARSq than the CART data mining algorithm.

## 4. Discussion

### 4.1. Pearson’s Correlation Coefficients

Animal live body weight is an important quantitative trait of economic value to the resource-poor farmer as sales and husbandry practices are highly dependent on it [6]. BW and linear body measurements are known to be highly correlated, a phenomenon used in developing BW prediction equations. Furthermore, interrelationships and correlations between BW and other linear body measurements are key in the selection and concurrent improvement of traits in genetic improvement programs [23]. The high, significant (*p* < 0.05), and strong correlations between live BW and HG in Tswana rams, ewes, and castrates found in this study have also been reported in the literature [24,25]. Such strong high correlation coefficients between live BW and other linear body measurements imply that such traits would be affected by changes in BW. That is, improving heart girth will concurrently improve body weight and, hence, HG can be used as an indirect selection criterion to improve live weight [24].

### 4.2. MARS Model

The MARS results indicated that HG was used as the most important sole predictor of BW in all sexes of Tswana sheep. This finding is similar to that in [6], which reported that HG was the best explanatory variable used to predict body weight in Dorper sheep of South Africa. The MARS model suggests that the influence on the indigenous Tswana sheep’s body was 1.81 when HG was >84 cm. This means that farmers can select indigenous Tswana sheep with more than 84 cm of HG for breeding purposes to obtain an improvement of 1.81 kg in live BW per cm of HG.

### 4.3. MARS and CART Data Mining Algorithm

The prediction of live BW from linear body measurements has recently been at the core of most animal breeding investigations and has been explored in livestock species [3,12]. However, pertinent challenges remain. For instance, the estimation of body weight from linear body measurements depends on the high correlation between BW and the linear body measurements. However, correlations only give the association (direction and strength) between BW and other linear body measurements and do not stipulate the effect of linear body measurements on live BW [8,26]. In this regard, the use of data mining algorithms is gaining popularity in BW prediction studies to overcome multicollinearity problems. Accordingly, data mining algorithms have been employed in this study to determine the effect of linear body measurements on the BW of Tswana sheep and, ultimately, predict the live BW of mature indigenous Tswana sheep of Botswana. Secondly, the MARS and CART algorithms have been compared using the goodness of fit criteria to establish the algorithm with the best predictive performance.

The results indicate that the MARS algorithm generated a simple and easy-to-interpret equation with the greatest predictive performance using only the predictor HG, whereas the CART data mining algorithm, which, according to the cross-validation method, produced seven complexity parameters bearing six splits. Therefore, in essence, MARS proves to be more precise in prediction performance than the CART data mining algorithm. The findings of the current study are in close consonance with Faraz et al. [11], who reported that the MARS algorithm is the best predictor of body weight in Pakistan Thali sheep with a goodness of fit of R^2^ = 0.90, Adj. R^2^ = 0.89, SD ratio = 0.312, and r = 0.95. Phaladi et al. [6] also pointed out that MARS was more informative in its predictive accuracy of BW in South African Dorper sheep compared to CART, Chi-squared automatic interaction detection (CHAID), and exhaustive CHAID (Ex-CHAID) algorithms. Similarly, Hlokoe [21] found the highest of R^2^ = 0.993, Adj. R^2^ = 0.991, SD ratio = 0.081, and lowest RSME = 5.97, validating that the MARS algorithm had more predictive performance for body weight in South African Nguni cattle compared to the CART model. Bila et al. [5] also recommended the MARS model over CART for the prediction of live BW in South African Sussex cattle. Again, MARS has also been recommended for different applications, including the prediction of hot carcass weight of cattle breeds [27]. Komadji et al. [28] also compared the effective predictiveness of MARS, CART, and SVR algorithms to predict peak particle velocity (PPV) in open-cast mines and found that the MARS model outperformed other models in that study, with lower overall scores (RMSE of 0.227 and R^2^ of 0.951).

On the other hand, in a recent study by Mathapo et al. [29], it was reported that the CART was the best predicting model for the prediction of body weight from the morphological traits of Nguni goats of Limpopo, South Africa. This is contrary to the findings of the current study. Furthermore, Matvieiev et al. [30] suggested that, as opposed to MARS, CART data mining displayed a better BW predictive performance than Ukrainian beef cattle. The findings of the current study are also contrary to the reports of Celik et al. [31] and Tirink et al. [32], who compared various data mining and machine learning algorithms and found that the CART algorithm was more reliable than MARS in predicting the body weight of the Mengali rams of Pakistan and Romane sheep, respectively. Tirink et al. [7] also predicted the birth weight of Morkaraman lambs from linear body measurements using data mining algorithms and recommended the adoption of the CART data mining algorithm due to a high coefficient of determination. Similarly, as per the goodness of fit test criteria, Assan et al. [33] suggested that the CART model outperformed the MARS model in predicting body weight in three genotypes of chicken in Zimbabwe. The results of the current study are further contrary to Mathapo et al. [34], who concluded that the CART model was a more effective model to predict BW in Bapedi sheep compared to MARS, based on a goodness of fit test. Marco et al. [35] also compared several machine learning algorithms and echoed that the CART algorithm was more usable. These discrepancies in results may be caused by the data and sample size.

## 5. Conclusions

The findings of this study showed a positive correlation between BW and other biometric traits, with the highest correlation observed between BW and HG across all sexes. Additionally, MARS demonstrated that HG significantly influences the BW of Tswana sheep. This suggests that HG can serve as the sole predictor of BW in the indigenous sheep of Botswana. The goodness of fit test criteria indicated that the MARS data mining algorithm offers higher predictive performance than the CART algorithm and even the simple linear regression model. Furthermore, although the algorithms can predict live BW, the MARS algorithm produced a simpler and more interpretable equation with the greatest predictive performance, utilizing only the predictor HG, in contrast to the CART data mining algorithm, which, according to the cross-validation method, proved to be more complex and less precise. This study’s findings will aid farmers in predicting BW in Tswana sheep.

## Figures and Tables

**Table 1:** Overall descriptive statistics of biometric traits in Tswana sheep.

Trait		Rams			Ewes	
	Mean ± SD	Min	Max	Mean ± SD	Min	Max
BW (kg)	43.20 ± 0.55	26.00	73.00	36.95 ± 0.35	20.00	59.00
HG (cm)	80.56 ± 0.44	65.00	99.00	75.41 ± 0.28	60.00	91.00
BL (cm)	62.67 ± 0.46	52.00	80.00	61.28 ± 0.29	32.00	75.00
SW (cm)	22.51 ± 0.20	17.00	31.00	20.59 ± 0.13	14.00	28.00
NL (cm)	31.65 ± 0.28	22.00	40.00	29.90 ± 0.18	18.00	38.00
WH (cm)	67.50 ± 0.34	52.00	79.00	63.23 ± 0.21	46.00	73.00
RH (cm)	66.64 ± 0.33	54.00	80.00	63.47 ± 0.21	42.00	73.00
HW (cm)	12.33 ± 0.11	8.00	17.00	10.45 ± 0.07	7.00	16.00
HL (cm)	16.92 ± 0.18	10.00	26.00	15.31 ± 0.11	9.00	27.00
EW (cm)	6.12 ± 0.07	4.00	8.00	5.83 ± 0.04	3.00	8.00
EL (cm)	11.87 ± 0.12	9.00	16.00	11.97 ± 0.07	8.00	16.00
CBC (cm)	7.79 ± 0.23	6.00	9.00	7.19 ± 0.14	5.00	10.00
CBL (cm)	15.98 ± 0.12	13.00	20.00	15.20 ± 0.08	11.00	18.00
RL (cm)	25.23 ± 0.32	14.00	33.00	23.44 ± 0.20	14.00	30.00
RW (cm)	17.33 ± 0.16	13.00	24.00	16.76 ± 0.10	12.00	22.00
TL (cm)	37.09 ± 0.41	26.00	49.00	34.56 ± 0.26	21.00	51.00
TC (cm)	21.75 ± 0.42	11.00	35.00	17.27 ± 0.27	8.50	37.00
SC (cm)	26.75 ± 0.23	16.00	32.00	N/A	N/A	N/A

BW = body weight, HG = heart girth, BL = body length; SW = shoulder width; NL = neck length; WH = wither height; RH = rump height; HW = head width; HL = head length; EW = ear width; EL = ear length; CBC = cannon bone circumference; CBL = cannon bone length; RL = rump length; RW = rump width; TL = tail length; TC = tail circumference; SC = scrotal circumference; N/A = not applicable; Min = minimum; Max = maximum; SD = standard deviation.

**Table 2:** Least squares, means, and standard errors for the effect of sex on biometric traits of indigenous Tswana sheep.

Trait	Rams	Ewes	Castrates
BW (kg)	43.20 ± 0.55 ^a^	36.95 ± 0.35 ^b^	42.34 ± 0.63 ^a^
HG (cm)	80.56 ± 0.44 ^a^	75.41 ± 0.28 ^b^	80.62 ± 0.41 ^a^
BL (cm)	62.67 ± 0.46	61.28 ± 0.29	63.04 ± 0.40
SW (cm)	22.51 ± 0.20 ^a^	20.59 ± 0.13 ^b^	22.66 ± 0.23 ^a^
NL (cm)	31.65 ± 0.28	29.90 ± 0.18	32.00 ± 0.25
WH (cm)	67.50 ± 0.34 ^a^	63.23 ± 0.21 ^b^	66.79 ± 0.32 ^a^
RH (cm)	66.64 ± 0.33 ^a^	63.47 ± 0.21 ^b^	66.43 ± 0.27 ^a^
HW (cm)	12.33 ± 0.11 ^a^	10.45 ± 0.07 ^b^	12.43 ± 0.14 ^a^
HL (cm)	16.92 ± 0.18 ^a^	15.31 ± 0.11 ^b^	16.01 ± 0.22 ^a^
EW (cm)	6.12 ± 0.07	5.83 ± 0.04	5.98 ± 0.05
EL (cm)	11.87 ± 0.12	11.97 ± 0.07	11.91 ± 0.15
CBC (cm)	7.79 ± 0.23 ^a^	6.88 ± 0.14 ^b^	7.89 ± 0.21 ^a^
CBL (cm)	15.98 ± 0.12	15.20 ± 0.08	15.78 ± 0.15
RL (cm)	25.23 ± 0.32 ^a^	23.44 ± 0.20 ^b^	24.67 ± 0.41 ^a^
RW (cm)	17.33 ± 0.16 ^a^	16.76 ± 0.10 ^b^	17.67 ± 0.21 ^a^
TL (cm)	37.09 ± 0.41 ^a^	34.56 ± 0.26 ^b^	39.13 ± 0.36 ^a^
TC (cm)	21.75 ± 0.42 ^a^	17.27 ± 0.27 ^b^	23.61 ± 0.39 ^a^
SC (cm)	26.75 ± 0.23	N/A	N/A

BW = body weight, HG = heart girth, BL = body length; SW = shoulder width; NL = neck length; WH = wither height; RH = rump height; HW = head width; HL = head length; EW = ear width; EL = ear length; CBC = cannon bone circumference; CBL = cannon bone length; RL = rump length; RW = rump width; TL = tail length; TC = tail circumference; SC = scrotal circumference; N/A = not applicable; ^ab^ Means within a row bearing different superscripts are significantly different (*p* < 0.05); ^a^ is assigned to the highest value.

**Table 3:** Correlation coefficients between BW and biometric traits of Tswana sheep in different agroecological regions of Botswana (above diagonal for rams and below diagonal for ewes).

	BW	HG	BL	SW	NL	WH	RH	HW	HL	EW	EL	CBC	CBL	RL	RW	TL	TC
BW		0.99 **	0.83 **	0.72 **	0.53 **	0.80 **	0.85 **	0.38	0.49 *	0.12	0.095	0.09	0.46 *	0.66 **	0.52 **	0.21	0.20
HG	0.99 **		0.82 **	0.76 **	0.55 **	0.81 **	0.85 **	0.36	0.50 *	0.10	0.089	0.030	0.50 *	0.66 **	0.53 **	0.21	0.17
BL	0.79 **	0.64 **		0.52 **	0.33	0.57 **	0.70 **	0.22	0.30	0.14	0.028	0.020	0.41 *	0.45 *	0.35	0.01	0.01
SW	0.66 **	0.49 *	0.81 **		0.57 **	0.66 **	0.53 **	0.51 *	0.51 *	−0.16	−0.11	−0.019	0.60 **	0.53 **	0.60 **	0.20	−0.01
NL	0.58 **	0.38	0.60 *	0.45 *		0.48 *	0.44 *	0.71 **	0.41 *	0.042	−0.014	−0.33	0.30	0.48 *	0.30	0.50 *	0.09
WH	0.80 **	0.73 *	0.44 *	0.27	0.31		0.90 **	0.34	0.48 *	0.22	0.16	0.002	0.52 **	0.78 **	0.61 **	0.42 *	0.12
RH	0.77 **	0.67 **	0.50	0.37	0.27	0.66 **		0.22	0.36	0.36	0.13	−0.030	0.46 *	0.81 **	0.58 **	0.34	0.23
HW	0.23	0.44 *	0.38	0.34	0.22	−0.29	0.34		0.52 **	−0.08	0.13	−0.22	0.26	0.21	0.20	0.40	−0.32
HL	0.38	0.45 *	0.46 *	0.22	0.72 **	0.32	0.37	0.19		−0.04	0.12	−0.04	0.26	0.15	0.016	−0.01	−0.11
EW	0.091	0.38	0.22	0.45 *	0.34	0.22	−0.12	−0.019	−0.22		0.75 **	0.044	0.23	0.25	−0.024	0.30	0.16
EL	0.19	−0.20	0.17	0.38	−0.28	−0.023	−0.28	0.12	0.23	0.76 **		0.21	0.17	−0.13	−0.27	0.13	−0.17
CBC	0.10	0.11	−0.21	−0.13	0.23	0.44 *	0.27	0.14	−0.38	−0.20	−0.012		−0.004	−0.14	−0.033	−0.16	−0.07
CBL	0.38 *	0.27	0.10	0.23	0.37	0.29	0.31	0.19	−0.41	0.34	0.23	0.20		0.51 *	0.48 *	0.25	−0.04
RL	0.57 **	0.33	0.22	0.27	0.19	0.092	0.24	0.34	0.29	−0.23	−0.14	0.23	−0.29		0.80 **	0.50 *	0.43 *
RW	0.61 **	0.56 *	0.39	0.22	0.42	0.51 *	0.70 *	0.26	0.19	−0.27	−0.22	0.30	0.27	0.41 *		0.40	0.17
TL	0.18	0.31	0.22	0.32	0.28	0.19	0.32	−0.098	0.21	0.29	0.15	0.21	−0.67	−0.33	−0.56		0.40
TC	0.11	0.19	0.19	0.16	0.22	0.34	0.24	0.33	0.36	0.21	−0.021	0.32	−0.09	0.06	0.15	0.42 *	

BW = body weight, HG = heart girth, BL = body length; SW = shoulder width; NL = neck length; WH = wither height; RH = rump height; HW = head width; HL = head length; EW = ear width; EL = ear length; CBC = cannon bone circumference; CBL = cannon bone length; RL = rump length; RW = rump width; TL = tail length; TC = tail circumference. ** = significant at *p* < 0.001; * = significant at *p* < 0.05.

**Table 4:** Correlation coefficients between BW and biometric traits of Tswana sheep castrates in different agroecological regions of Botswana.

	BW	HG	BL	SW	NL	WH	RH	HW	HL	EW	EL	CBC	CBL	RL	RW	TL	TC
BW		0.99 **	0.77 **	0.58 **	0.12	0.32	0.41	0.01	0.20	0.35	0.25	0.41	0.56 *	0.57 **	0.63 **	0.20	0.44 *
HG			0.78 **	0.58 **	0.08	0.32	0.42 *	−0.02	0.18	0.34	0.22 ^ns^	0.42 *	0.55 **	0.59 **	0.62 **	0.20	0.43 *
BL				0.55 **	0.31	0.29	0.42 *	0.27	0.15	0.10	0.11	0.39	0.42 *	0.46 *	0.54 **	−0.01	0.38
SW					0.42 *	0.31	0.40	0.04	0.54 **	0.16	0.06	0.41 ^ns^	0.52 *	0.48 *	0.61 **	0.23	0.34
NL						0.52 *	0.40	0.34	0.62 **	0.08	0.42 *	0.42 *	0.56 **	0.16	0.45 *	0.30	0.38
WH							0.89 **	0.05	0.49	0.25	0.45 *	0.20	0.77 **	0.44 *	0.63 **	0.59 **	0.22
RH								−0.11	0.40	0.19	0.18	0.18	0.65 **	0.57 **	0.67 **	0.42 *	0.33
HW									0.14	−0.11	0.41	0.23	0.05	−0.25	0.10	0.15	0.02
HL										0.11	0.44 *	0.50 *	0.72 **	0.16	0.41	0.33	0.39
EW											0.48 *	−0.01	0.40	0.61 **	0.50 *	0.45 *	0.20
EL												0.33	0.54 **	0.05	0.23	0.46 *	0.11
CBC													0.55 **	0.12	0.24	−0.09	0.41
CBL														0.45 *	0.71 **	0.54 **	0.43 *
RL															0.80 **	0.46 *	0.39
RW																0.42 *	0.61 **
TL																	0.08
TC																	

BW = body weight, HG = heart girth, BL = body length; SW = shoulder width; NL = neck length; WH = wither height; RH = rump height; HW = head width; HL = head length; EW = ear width; EL = ear length; CBC = cannon bone circumference; CBL = cannon bone length; RL = rump length; RW = rump width; TL = tail length; TC = tail circumference. ** = significant at *p* < 0.001; * = significant at *p* < 0.05. ns = not significant.

**Table 5:** Multivariate adaptive regression splines model.

Variables	Coefficients
Intercept	46.32
h (84-HG)	−1.11
h (HG84)	1.81

h = hinge function; HG = heart girth.

**Table 6:** Best-fitted regression models with stepwise regression analysis on morphological traits of Tswana sheep.

Estimator	Model	RMSE	Rsq	CV	F-Value
Rams					
HG	BW = −60.14 + 1.29HG	1.43	0.976	3.37	5581.33 **
HG + BL	BW = −68.43 + 0.65HG + 0.94BL	1.04	0.987	2.46	5288.24 **
HG + BL + SW	BW = −58.89 + 0.53HG + 0.73BL + 0.61SW	0.98	0.989	2.31	4031.80 **
HG + BL + SW + WH	BW = −58.5 + 0.53HG + 0.73BL + 0.62SW − 0.02WH	0.978	0.989	2.31	3002.07 **
Ewes					
HG	BW = −49.86 + 1.15HG	0.744	0.989	1.93	33041.2 **
HG + BL	BW = −49.71 + 1.10HG + 0.06BL	0.741	0.989	1.92	16668.1 **
HG + BL + WH	BW = −50.79 + 1.09HG = 0.03BL + 0.06WH	0.741	0.989	1.92	11105.3 **
Castrates					
HG	BW = −60.10 + 1.28HG	1.78	0.962	4.22	3517.47 **
HG + BL	BW = −66.55 + 0.70HG + 086BL	1.40	0.977	3.31	2889.75 **
HG + BL + RW	BW = −55.87 + 053HG + 0.61BL + 1.04RW	1.22	0.983	2.88	2561.89 **

BW = body weight; HG = heart girth; BL = body length; SW = shoulder width; WH = wither height; RH = rump height; RMSE = root-mean-square error; CV = coefficient of variation; Rsq = coefficient of determination. ** = significant at *p* < 0.001.

**Table 7:** CART algorithm results based on the cross-validation method.

Complexity	Parameter	Number of Splits	Relative Error	Mean of the Error
1	0.61	0	1.00	1.00
2	0.17	1	0.38	0.39
3	0.09	2	0.21	0.22
4	0.03	3	0.12	0.12
5	0.03	4	0.09	0.12
6	0.02	5	0.06	0.08
7	0.01	6	0.04	0.05

**Table 8:** Goodness of fit criteria showing the predictive performance of the MARS and CART algorithms.

	MARS		CART	
Criterion	Training	Test	Training	Test
RMSE	0.476	0.442	1.611	1.917
RRMSE	1.200	1.095	4.059	4.475
SDR	0.060	0.049	0.209	0.212
CV	1.200	1.100	4.060	4.750
R	0.998	0.999	0.978	0.977
PI	0.601	0.548	2.052	2.400
ME	0.000	0.021	0.000	−0.009
RAE	0.000	0.000	0.002	0.002
MAPE	0.975	0.860	3.465	3.690
MAD	0.371	0.329	1.302	1.454
Rsq	0.996	0.998	0.956	0.995
ARSq	0.996	0.998	0.956	0.995
AIC	−669.26	−307.34	433.89	249.89

RMSE = root-mean-square error; RRMSE = relative root-mean-square error; SDR = standard deviation ratio; CV = coefficient of variation; r = Pearson’s correlation coefficient; PI = performance index; ME = mean error; RAE = relative approximation error; MAPE = mean absolute percentage error; MAD = mean absolute deviation (MAD); Rsq = coefficient of determination; ARSq = adjusted coefficient of determination; AIC = Akaike’s information criterion.

## Data Availability

The data are contained within the article.
